# Newborn screening for SCID: the very first prospective pilot study from Türkiye

**DOI:** 10.3389/fimmu.2024.1384195

**Published:** 2024-10-02

**Authors:** Sule Haskologlu, Senem Kocak, Lale Satiroglu Tufan, Fethiye Eken Aksoy, Dilan Bastug, Deniz Aslar Oner, Candan Islamoglu, Kubra Baskin, Saliha Esenboga, Deniz Acican, Serdar Ceylaner, Sukru Nail Guner, Sevgi Keles, Deniz Cagdas, Ismail Reisli, Basak Tezel, Figen Dogu, Ilhan Tezcan, Aydan Ikinciogullari

**Affiliations:** ^1^ Department of Pediatrics, Division of Immunology and Allergy, School of Medicine, Ankara University, Ankara, Türkiye; ^2^ Department of Forensic Medicine, Forensic Genetics Laboratory, School of Medicine, Ankara University, Ankara, Türkiye; ^3^ Department of Pediatrics, Division of Immunology, School of Medicine, Hacettepe University, Ankara, Türkiye; ^4^ Pediatric and Adolescent Health Department, The Ministry of Health, Public Health General Directorate, Ankara, Türkiye; ^5^ Intergen Genetic and Rare Diseases Diagnosis Centre, Ankara, Türkiye; ^6^ Department of Pediatrics Division of Immunology and Allergy, Meram Faculty of Medicine, Necmettin Erbakan University, Konya, Türkiye

**Keywords:** newborn screening, TREC, SCID, Türkiye, dried blood sample (DBS)

## Abstract

**Purpose:**

The measurement of T-cell receptor excision circle (TREC) is used for newborn screening (NBS) in dried blood spot (DBS) samples from Guthrie card for severe combined immunodeficiency (SCID). Here, we report the results of first newborn screening pilot program for SCID conducted in Türkiye.

**Methods:**

The study was carried out together with Ankara University School of Medicine and The Ministry of Health, Public Health General Directorate, Pediatric and Adolescent Health Department. TREC measurements were performed in randomly selected Guthrie card samples obtained from 20253 babies born between October 2018 and October 2020. The TREC analyses were performed together with beta Actin (β-Actin) via RT-PCR (Real Time Polymerase Chain Reaction).

**Results:**

TRECs found to be normal (≥15 copies/µl) in 98,6% of the newborns (n: 19975) but low (<15 copies/µl) in 1.4% (n:278) at the initial analyses. TRECs were retested in 278 suspected infants and found to be normal in 160 (0.8%) while low in 118 (0.58%). New DBS were obtained from the babies with low TRECs (new sample test). TRECs were normal in 108 (0.53%) of the new sample tests and low in 10 (0.049%). Two among 10 babies who had abnormal (undetectable) TRECs were diagnosed as SCID; ADA (P1) and RAG1 (P2) defects were confirmed respectively. They both received curative treatments [gene therapy (P1) and HSCT (P2)]. The remaining 6 of 8 newborns with abnormal TRECs were found normal after clinical and laboratory immune work-up, while medical records of other two revealed early postnatal death due to extreme prematurity.

**Conclusion:**

In the light of this study the incidence of SCID was detected at least 1/10000 live births in Türkiye. This study shows the feasibility and usefulness of initiating SCID screening in Türkiye.

## Introduction

Severe Combined Immune Deficiency (SCID) is a primary immunodeficiency (PID) with the most critical natural progression leading to death in 1-2 years. It develops as a result of inborn errors of about 20 genes that are responsible for the development of T-lymphocytes that play a pivotal role in a healthy immune response (1).

SCID patients seem healthy at birth, but they generally seek medical advice with findings of infections within weeks to months. The common occurrence of infections in infancy makes the diagnosis difficult. If there has not been a similar case previously diagnosed in the family or if such an occurrence is missed when taking the family history, the diagnosis is further delayed. If these cases are diagnosed after life-threatening infections, organ damages, increased need for intensive care unit admissions and long-term hospital care, brings about higher treatment costs, and eventually leads to mortality. In cases of late diagnosis, also the administration of live vaccines especially BCG, increases morbidity and mortality. Curative treatment mostly includes hematopoietic stem cell transplantation (HSCT) or gene therapy (GT), but a significant minority has athymia, which requires a thymus transplant (1, 2). It was shown that SCID which was diagnosed and treated with HSCT in the first 3.5 months of life had improved survival and reduced morbidity (1). SCID meets the criteria for newborn screening (NBS) disease because affected infants are asymptomatic at birth, the disease is fatal without treatment, and the outcome improves significantly if early management is provided. In 2005, Chan and Puck published the first method for SCID screening that used PCR to extract TREC measurements from dried blood spots (3). Many screening programs around the world have screened newborns for SCID since it was first used in Wisconsin in 2008 (4). The SCID NBS programs based on measuring the molecular biomarker T-cell receptor excision circles (TRECs), which is produced by T-cell receptor gene recombination during thymocyte differentiation into mature T cells (5) TRECs deficiency indicates severe T-cell lymphopenia. By means of a NBS via TREC examination of dried blood spots (DBS), the frequency of SCID was identified as 1/58000 in USA (6). This frequency is two fold higher compared to the previous data. In Navajo; a closed community in USA, the incidence is identified as 1/2000 (7). It is reported as 1/22500 in Israel and as 1/2906 in Saudi Arabia (8, 9). The incidence of SCID was reported as 1/63000 in France, 1/54000 in Germany, and 1/45000 in Sweden (10–12).

In Türkiye, the incidence of SCID is not clear. SCID incidence was previously reported as 1/10000 in Konya province via a retrospective study (13). However, according to our experts’ opinions, SCID is quite a common entity that constitutes a public health problem that remains hidden since most of the cases in our country die from infectious causes before being diagnosed.

This prospective pilot study aimed to perform NBS via TREC analysis for the first time to achieve early diagnosis and estimate the incidence of SCID in Türkiye. Thus, the patients will be rapidly diagnosed at an early stage and effective treatment will be provided.

## Materials and methods

### Generation of samples for the TREC test

The study was carried out as a subproject (one out of three projects) of a joined The Scientific and Technological Research Council of Turkey (TUBITAK) 1003 project under the collaboration between Ankara University School of Medicine and The Ministry of Health, Public Health General Directorate Pediatric, Adolescent Health Department. Approval for the study was obtained from the Hacettepe University Clinical Research Ethics Committee (approval number: KA-15069). TREC levels were measured in 20253 newborns who were randomly selected from all those born in Ankara and Konya provinces between October 2018 and October 2020. The number of live births in the two provinces in two-year period is 210000. So in our study, approximately 10% (20253 newborns in two years =9.6%) of all births were screened. Dried blood samples were obtained on Guthrie cards collected in the ordinary routine NBS program (NBSP) within the first 48 hours of life and were sent to either of the two central NBS laboratories (located in Ankara and Istanbul) of the Ministry of Health Public Health General Directorate. The study group visited the NBS Laboratory in Ankara, obtained 3 discs of 3.2 mm from Guthrie card DBS, and placed them in 1.5 ml Eppendorf tubes.

The study samples were assigned a unique barcode and labelled in our laboratory. Information regarding birth weight (BW), gestational week (GW), delivery route, and gender of the babies was recorded from the cards.

### DNA isolation

Three 3.2 mm discs, obtained from each Guthrie card, were used for DNA isolation. Magnesia 16 Tissue Genomic DNA Extraction Kit (96 strips/Box-401; Catalog no: AE4012) and robotic isolation device of Anatolia Gene Works Magnesia (Mg160079) were found as the most relevant and optimized protocol for the isolation of qualified amount of DNA from DBS discs. As a quality control, ACTB amplification was used to assess the success of DNA extraction from the Guthrie cards. After DNA isolation samples were used immediately and also stored at -20°C for further need of repeated studies.

### Real time-polymerase chain reaction

For the multiplex TREC and β-Actin rt-PCR assay; master mix was prepared, including 0.5 mmol/L Forward/Reverse primers and 0.15 mmol/L TaqMan probes for both TREC and β-Actin, 1Xpolymerase (Luna^®^ Universal Probe qPCR Master Mix Catalog #: M3004S) and dH2O. A total of 5 uL DNA extract was used for each sample. The reactions were carried out on a Roche LightCycler^®^ 480 Instrument, 96-well (Roche, Germany) and all analyzed RT-qPCR assays fulfilled the quality requirements. DNA sequence of the primers and probes previously published as shown in **Supplementary Table 1** (4, 14, 15) for the multiplex TREC/β-Actin assay was used. Calibration curves were created by 10-fold serial dilution of plasmids containing TREC and β-Actin sequences. By taking plasmid copy numbers as a reference, LightCycler software was used to identify the copy numbers corresponding to the Cp value of each sample. TREC measurements were performed according to the in-house assay (not a commercially avaiable kit) that previously constituted via a retrospective study funded by Ankara University’s Scientific Research Projects (BAP no: 16A0230008) and and to the values reported in literature (4, 15–17). In 2015 and 2016, TREC was measured in an anonymous sample group of 5030 newborns from DNA samples from dried blood on Guthrie cards. TRECs value > 46 copies/DBS were regarded as normal, ≤ 46 copies/DBS as lowIn the literature, each disc is regarded to contain 3 microliters (µl) of blood (18). From this point of view, TREC levels were normalized per microliter of blood, assuming that the sample contains approximately 3 uL of whole blood, TREC (46 copies/DBS) cut-off would correspond to 15 copies/µl. So, the cutoff value for TREC and β-Actin copy numbers were identified as 15 copies/µl and 1233 copies/µl, respectively. Peripheral blood samples from 100 healthy blood bank volunteers aged between 18 and 65 years and DBS from 10 patients previously diagnosed with SCID were used as internal positive controls. TRECs values were detected as 0 in all SCID cases.

In our study, due to difficulties in obtaining commercial kits which have been used in many countries where SCID has been screened (6–12). We used a homemade assay that was developed and performed by Azzari et al. in Tuscany (personal communication via onsite visit). This method was adapted and used in our laboratory both through the retrospective study and this prospective study (4, 15–17).

The terminology of the algorithm was used in agreement with a recent recommendation for uniform standardization of TREC-NBS terminology (19).

### TREC screening algorithm

**Figure 1:**
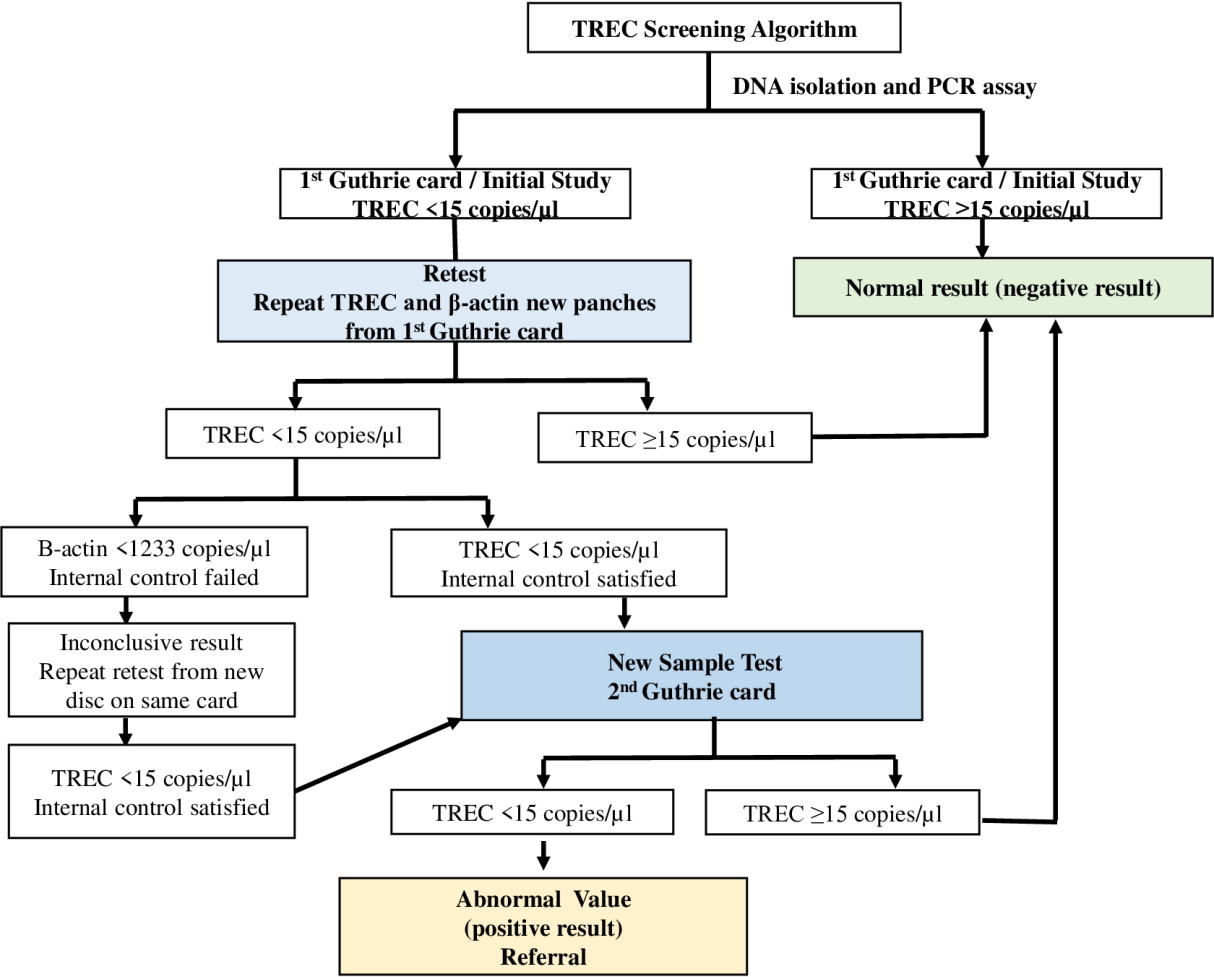
The flow chart of the study.

For the initial study, a TREC copy number above the cutoff value of ≥15 was considered normal; one below 15 was considered abnormal. If TREC values were abnormal, retest was performed from the same Guthrie card discs. If TRECs again found as abnormal, new samples were obtained from a second Guthrie card and the measurements were repeated on these new samples. In Turkish NBSP, the second Guthrie card samples are routinely taken between the 48^th^ hours and first week of life. If any abnormal values were detected during the new sample test, they were referred to the centers conducting the study for confirmation. The flow chart of the study is shown in **Figure 1**.

### Cost of TREC measurement

The whole assay including DNA isolation and RT-PCR is found to be $5.1 per test. This study is actually not a cost-effectiveness study. However, our retrospective cost-effectiveness study (20) allowed us to compare the costs of early versus late diagnosis in SCID patients previously.

### Statistics

Statistical analyses were performed on the SPSS software (IBM Corp. Released 2018, IBM SPSS Statistics for Windows, Version 20, NY, USA). A descriptive analysis was carried out. Following the application of the Kolmogorov–Smirnov test, the data were not distributed normally and were therefore shown as median and Interquartile Range (IQR), respectively, or by the minimum and maximum scores in the assay. Comparison of TREC concentrations between the demographic characteristics of newborns was established using the Independent Sample Mann-Whitney U test.

## Results

### Evaluated newborns, repeated test frequencies and outcome

**Figure 2:**
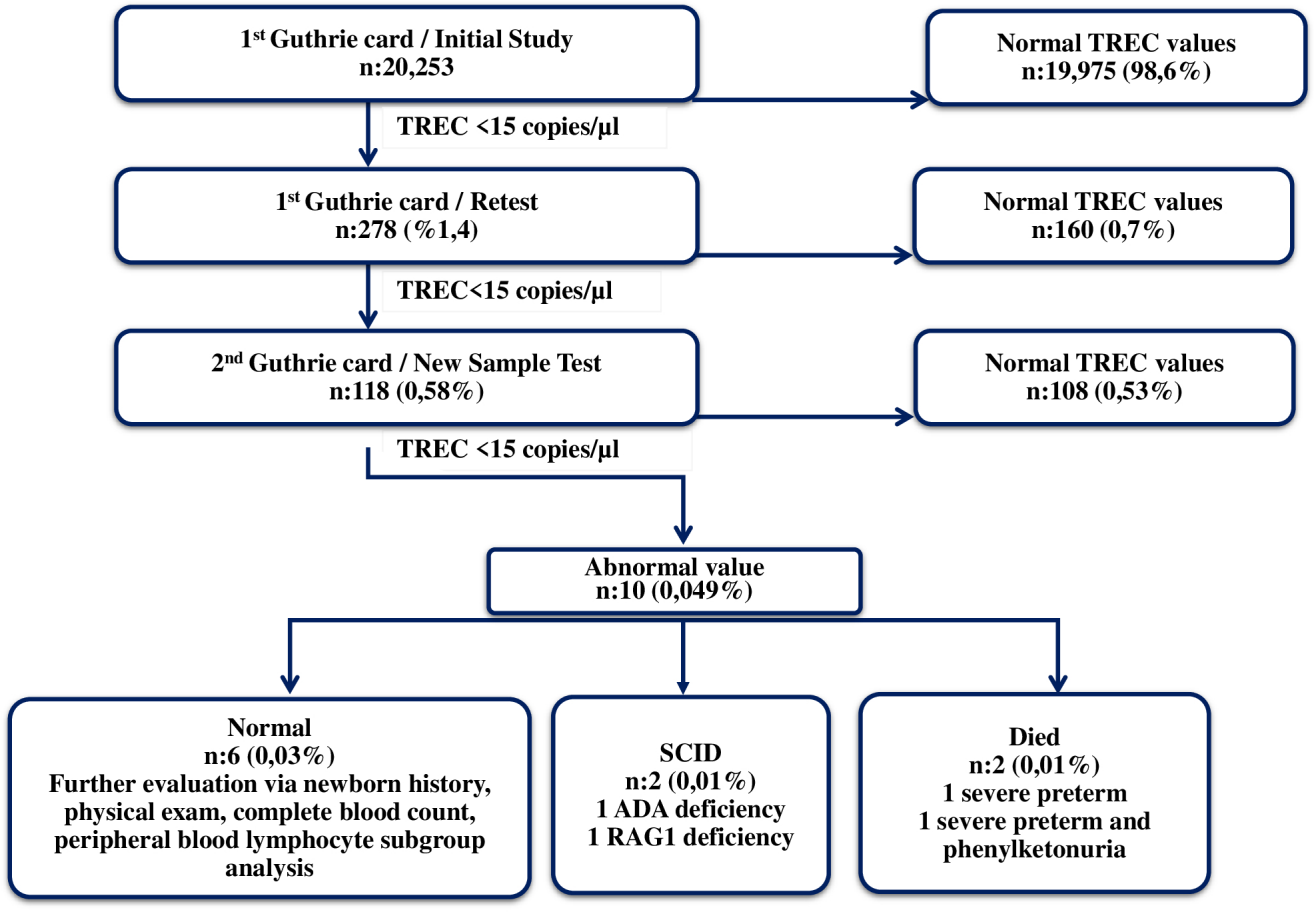
The evaluation of the results and outcome.

TREC measurements were carried out on randomly selected Guthrie card samples obtained from 20,253 babies born between October 2018 and October 2020. The median TREC and β-Actin values were 264 (min-max: 0-16024) and 18300 (min-max: 1120-117500), respectively. Samples with TREC values ≥ 15copies/µl were considered normal, while those with TREC values < 15 copies/µl were reported to Newborn Screening Laboratories as having an “abnormal value”. The initial TREC test results were obtained on average nine days after the babies were born. TREC values were found to be normal in 98.6% of the newborns (n:19975) on the initial measurement, whereas 1.4% (n:278) had abnormal values. The TREC values were normal in 160 babies (0.8%), and abnormal in 118 (0.58%) of retested samples (1.4% retest rate). New samples were obtained from these infants with abnormal TREC using the new Guthrie cards (new sample test). In the measurements conducted on the new samples 108 (0.53%) had normal and 10 had (0.049%) abnormal TREC. These samples were suspected to be T-cell lymphopenia (TCL) and were referred for additional testing (referral rate was 0.049%). Among these 10 newborns, TREC measured as 0 copy/µl in two babies. These two babies were reported to the Ministry of Health, Public Health General Directorate, Newborn Screening Laboratories with the suspicion of SCID. One of the babies was born in Ankara and the other in Konya. Both of them were examined and evaluated by expert immunologists who are working at the project centers in the cities where they lived. After obtaining their family histories, and performing physical examination, complete blood count (CBC) and peripheral blood lymphocyte subgroup (PBLS) measurements were performed. P1 had marked lymphopenia (ranged between 240 and 380/mm3) which was detected at the initial and subsequent CBC. The PBLS analyses demonstrated extremely low CD3+ T cells (26/mm^3^), CD4+ T cells (12/mm^3^), CD8+ T cells (14/mm^3^), CD19+ B cells (0/mm^3^) and NK cells (89/mm³). The results were in consistent with T-B-NK+ SCID. P2’s absolute lymphocyte counts also found to be very low with a range of 230-620/mm³. In the PBLS analysis, no CD3+, CD4+, CD8+ or CD19+ lymphocytes were detected. Instead, all lymphocytes were identified as NK cells. The immunophenotype of P2 was also assessed as T-B-NK+ SCID. Unfortunately, P1 had infection (interstitial pneumonia) when we confirmed the SCID diagnosis. While, P2.did not have any SCID related entity. Early diagnosis via NBS served as a lifesaving procedure; allowing us to initiate timely supportive treatments (like Ig replacement, ADA enzyme), prophylactic antimicrobials and avoidance of live vaccines (like BCG). So NBS allowed them to reach the curative treatments without developing any further infections, organ damages or BCG vaccine-related complications. Genes responsible for the disease were identified through genetic analysis. The first baby had ADA deficiency, and the second had RAG1 deficiency. The first patient underwent GT at 17 months of age in Milan, Italy, and the second received HSCT at 9 months of age from a fully HLA-matched healthy twin sibling donor at our center in Ankara. The Ministry of Health covered all the costs of curative treatments for both cases. After successful curative treatments, both patients experienced significant immune reconstitution. P1 is 5,5 years old at the moment with a stable clinical status and immune reconstitution. He has no physical or neurological problems except for a slight speech delay. No hospitalization required, and no other complications related to the GT were observed. He is off enzyme replacement in addition of being free of Ig replacement. P2 is now 4.5 years old. Her post-transplant T, B cell counts, lymphocyte functions and RTE levels reached to age-matched normal for healthy Turkish children (21, 22). She is free of Ig replacement with 93% donor T cell chimerism. No serious infections or HSCT-related complications have been observed after HSCT. Post-transplant vaccination programme is completed. The detailed clinical characteristics of the patients, along with the immunological evaluations conducted at the time of diagnosis, following treatments, and the outcomes are presented in **Table 1**. Eight other babies who had abnormal TREC values, two of which died due to extremely premature births and one having phenylketonuria as shown in **Figure 2**. The remaining six infants who had low TRECs (12 to 14 copies/µl) were evaluated by expert immunologists. The family histories, physical examinations, CBC results, and PBLS levels were normal. They were identified as false positives. The immune work-up of these 6 infants with low TREC detected at initial NBS were given in **Supplementary Table 2**. Results showed an incidence of SCID of at least 1/10000 live births in Türkiye. The false positive rate was 0.039% (8/20253).

**Table 1:** Clinical features, immunologic evaluation, treatment and outcome of patients diagnosed SCID via NBS by TREC measurement.

Patient 1 (P1)									Patient 2 (P2)					
**Baby** **Boy**	**GW:** 38 w		**Symptoms**	**Onset of symptoms (day)**			**SCID-related infection**	**SCID diagnosis by FC (day)**	**Baby Girl**	**GW:** 37 w	**Symptoms**	**Onset of symptoms (day)**	**SCID-related infection**	**SCID diagnosis by FC (day)**
	**BW:** 2000 gr		Fever and cough	15			Interstial Pneumonia	22		**BW:** 2060 gr	A single pustular lesion behind ear	21	No	30
	**Consanguinity: +**									**Consanguinity: +**				
Longitudinal immunological investigations and immune reconstitution following the curative treatment														
	**Before GT**				**After GT**				**Before HSCT**		**After HSCT**			
	**16^th^** **day of age**	**18^th^** **day of age**	**22^nd^** **day of age**	12 months of age^**^	**1^st^ year (at 29 mo)**	**2^nd^year (at 41mo)**	**3^rd^ year** **(53 mo)**	**4^th^ year (65 mo)**	**30^th^** **day of age**	**5 months of age**	**1^st^ year** **(21mo)**	**2^nd^ year (33 mo)**	**3^rd^ year** **(45 mo)**	**Healthy control (3-5 years)**
**Hb (g/dl)**	16.6	13.7	13.3	13,1	13	14.4	15.1	14.4	10.9	9.1	11.9	11.7	11.5	11-14
**WBC (mm^3^)**	2890	12400	17.920	3050	1930	3530	3240	9680	8.000	4960	4800	5910	6580	5500-14500
**ANC (mm^3^)**	1760	7440	11.290	2510	900	1730	1760	4870	5.700	2340	2240	4000	4050	1500-8000
**ALC (mm^3^)**	290	380	240	140	240	1040	870	3700	620	230	1860	1360	1680	1500-5500
**AEC (mm^3^)**	30	430	500	140	610	70	70	70	720	520	260	180	220	0-500
**Plt (mm^3^)**	315000	173000	91000	420000	291000	279000	259000	227000	325.000	473000	289000	149000	275000	150000-450000
**IgG (mg/dl)**	nt	nt	340	771	918	906	914	920	360	1200	321	505	709	745-1804
**IgA (mg/dl)**	nt	nt	<6	16	19	46	48	70	<6	<6	38	29	112	57-282
**IgM (mg/dl)**	nt	nt	<5	27	9	17	30	21	<5	<5	14	138	93	78-261
PBLS analyses, absolute counts, mm^3^ (%)														
**CD3+16-56-**	nt	nt	26 (11)	86 (59)	148 (62)	852 (82)	592 (68)	3000(81)	2 (0.3)	0	1376 (74)	1006 (74)	1108(66)	1900-3600 (55-79)
**CD3+CD4+**	nt	nt	12 (5)	50 (35)	67 (28)	145 (14)	183 (21)	407 (11)	1 (0.1)	0	967 (52)	734 (54)	722 (43)	600-2000 (26-49)
**CD3+CD8+**	nt	nt	14 (6)	32 (22)	62 (26)	353 (34)	305 (35)	1998(54)	1 (0.1)	0	390 (21)	245 (18)	353 (21)	300-1300 (9-35)
**CD3-16+56+**	nt	nt	214 (89)	10 (7)	24 (10)	62 (6)	61 (7)	148 (4)	608 (98)	207 (90)	279 (15)	95 (7)	269 (16)	200-1200 (5-28)
**CD19+**	nt	nt	0 (0)	43(30)	16 (7)	80 (7,7)	96 (11)	148 (4)	1 (0.1)	2 (1)	130 (7)	190 (14)	252 (15)	300-1200 (11-31)
**RTE**	nt	nt	0 (0)	nt	2,7 (4)	14 (10)	92 (20)	81 (20)	0	0	435 (60)	381 (52)	325 (45)	929-4981 (63-81)
Lymphocyte activation response to PHA and Anti CD3														
**CD3+CD25+**	nt	nt	0	nt	85	86	82	83	0,1	nt	86	84	73	52-94
**CD3+CD69+**	nt	nt	0	nt	88	86	84	81	0,2	nt	85	86	75	48-85
**CD4+CD25+**	nt	nt	0	nt	25	14	23	19	0,8	nt	56	63	47	–
**CD4+CD69+**	nt	nt	0	nt	25	14	22	19	0,5	nt	56	63	47	–
**Genetic defect**	c.956_960 del AAGAG (p.E319Gfs*3) (p.Glu319GlyfsTer3) a homozygous mutation in the ADA gene								RAG1 c. 1378 G>T (p.Gly460Ter)(p.G460*) a homozygous mutation in the RAG1 gene					
**Treatment**	IVIG, anti-microbial prophylaxis, ADA gene therapy at 16^th^ month of life								IVIG, anti-microbial prophylaxis, HSCT (MSD: twin brother at 9^th^ month of life)					
**Outcome**	48 mo after GT, alive and well, without Ig replacement, free from PEG-ADA enzyme,								45 mo after HSCT alive and well, without Ig replacement					

GW, Gestational Week; BW, Birth Weight; GT, Gene Therapy; HSCT, Hematopoetic Stem Cell Transplantation; FC, Flow Cytometry WBC, White Blood Count; ANC, Absolute Neutrophil Count; ALC, Absolute Lymphocyte Count; AEC, Absolute Eosinophil Count; RTE, CD4+CD45RA+CD31+; PHA, Phytohemagglutinin; nt, not tested.

**, Under PEG-ADA replacement therapy.

### Demographical features of newborns

Demographical features of the 20253 (gender, BW, GW, mode of delivery, and singleton/multiple pregnancy) were presented below and in the following **Table 2**. Of 20000 babies GW information was available 16200 (81%) was term (GW ≥38 weeks), 3800 was preterm (19%) (GW ≤37 weeks), median GW was 39 weeks (min-max: 21-43 weeks). The median TREC value of all preterm babies was 253 copies/µl, which was significantly lower than the median TREC value of term babies, which was 267 copies/µl (p<0.001). The median TREC values of late preterm babies (254 copies/µl) was significantly lower than median TREC values of term babies (267 copies/µl) (p<0.001).

**Table 2:** Gender, gestational week, birth weight, mode of delivery, number of babies and TREC copy number.

	**n (%)**	**TREC Copy number median (IQR)**	**P value**
**All newborns**	**20253 (100)**	**362 (187-440)**	
Gender			
Baby girl	10023 (49.7)	273 (265)	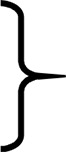 < 0.001
Baby boy	10135 (50.3)	255 (242)	
**Gestational week** (Median: 39 weeks, min-max: 21-43 weeks)	20000 (98.7)		
All Preterm (≤37 weeks)	3800 (19)	253 (250)	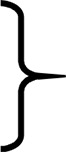 < 0.001 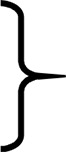 <0.001 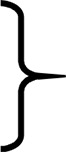 < 0.001 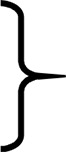 >0,05
Term (≥38 weeks)	16200 (81)	267 (254)	
Late preterm (32-≥37 weeks)	3718 (18.6)	254 (252)	
Very preterm (28-≥31 weeks)	68 (0.3)	199 (185)	
Severe preterm (≤27 weeks)	14 (0.1)	193 (405)	
**Birth weight** (Median: 3250 g, min-max: 500-5140 g)	20223 (99.8)		
Very low birth weight <1000 g	49 (0.2)	193 (267)	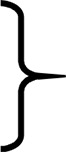 <0.001 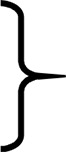 <0.001 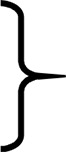 0,895
Low birth weight 1501-2500 g	1131 (5.6)	236 (206)	
Normal birth weight 2501-4000 g	18206 (89.8)	267 (254)	
High birth weight (big baby) ≥4001 g	837 (4.1)	266 (244)	
**Mode of delivery**	20130 (99.3)		
Vaginal	9429 (46.5)	264 (252)	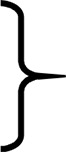 0.322
C-section	10701 (52.8)	266 (255)	
Number of babies			
Singleton	20090 (99.2)	264 (284)	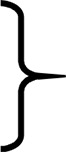 0.236
Multiple	163 (0.8)	252 (250)	

Although the TREC values decreased as the GW decreased, the difference in TREC value was found to be significant only between the late preterm (32-≥37 weeks) and very preterm (28-≥31) (p< 0.001). There wasn’t any significant difference between median TREC levels of very preterm (28-≥31 weeks) (199 copies/µl) and severe preterm (≤ 27 weeks) (193 copies/µl) (p>0.05). There was no significant difference between median TREC values late preterm babies (32-≥37 weeks) (254 copies/µl) and severe preterm babies (≤ 27 weeks) (193 copies/µl) (p>0.05).

BW information was available for 20223 babies, median BW was 3250 gr (min-max: 500-5410 g). There were 18206 newborns (90%) with normal BW, 1180 (5.8%) with low BW, and 837 (4.2%) with large BW. TREC values differed significantly between newborns with normal BW and newborns with low BW (p<0.001). Furthermore, TRECs changed substantially between newborns with low BW (1501-2500g) and very low BW (1500 g) (p=0.001).

When we assessed the retest and new sample test rates of term and preterm infants, we noticed that preterm had higher rates of both. The retest rate was 1.23% (n: 200/16200) and 2.02% (n: 77/3800) in term and preterm infants respectively. The new sample test rates were 0.51% (n: 83/16200) in term and 0.89% (n: 34/3800) in preterm infants. Five of ten samples with an abnormal value were term, while the other five were preterm. The rate of abnormal value was 0.13% in preterm and 0.03% in term.

## Discussion

SCID was the first immunodeficiency to be approved for population-based screening studies. These studies allowed the identification of the true incidence of SCID, led significant clinical benefits for affected infants, and lessons for public health, immunologists, and pediatricians (19, 23, 24). SCID has been screened in many countries for about 15 years, either through pilot studies or through national NBSPs. The incidence of SCID at the end of neonatal TREC screening varied among countries. However, the incidence of SCID has been reported to be higher than expected in many countries (6, 8–12, 19, 25). In this first prospective regional pilot TREC NBS study in Türkiye, conducted from October 2018 to October 2020 in 20253 babies born in Konya and Ankara provinces, the SCID incidence was identified as 1/10000. This incidence is consistent with the previously reported retrospective SCID incidence of 1/10000 identified in Konya (13). Our country has a higher incidence of SCID than United States, Europe, and Israel, but a lower incidence than Saudi Arabia. This is related to the frequency of consanguineous marriages in the societies and the autosomal recessive inheritance of the mutations in 18 of the 20 genes responsible for SCID. While the rate of consanguineous marriages in Türkiye is 23.2% (26), in Saudi Arabia it is 60% (9). We know that the SCID incidence of 1/10000 found in this regional pilot study doesn’t reflect the reality of our country. There is no doubt that the incidence of SCID will be much higher in a national NBSP. If we move to the eastern and south-eastern parts of Turkey, the prevalence of consanguineous marriages rises to 46.2% (26). The study’s most significant limitations are that it was only conducted in two provinces; central Anatolian neighboring cities Ankara and Konya and did not fully reflect the incidence and genetic diversity throughout the country. To obtain the most accurate results on these issues, nationwide screening is necessary. Although we are aware of this reality, we only screened the babies born in Konya and Ankara since we could conduct detailed evaluations to identify and treat suspected SCID cases rapidly. Türkiye’s population growth rate ranges from 0.8–1%, resulting the birth of at least 950000–1100000 babies every year (26). So, we can estimate that at least 100 babies with SCID born in Türkiye every year; quite higher compared to European countries and USA. Currently, some of these patients can be diagnosed due to family history and experienced doctors, while others unfortunately remain undiagnosed and died. So, the inclusion of SCID to the NBSP is even more necessary and carries a high priority for our country.

The state of California has the largest SCID-NBS series to date, 3252156 babies were screened from 2010 to 2017, and 50 were diagnosed with SCID. Forty-nine babies were followed up and 46 (94%) survived with allogeneic HSCT, GT, or enzyme replacement (27). The Primary Immune Deficiency Treatment Consortium (PIDTC) studied the outcomes of HSCT in children diagnosed with SCID by NBS or family history, and found that 95% of infection-free transplanted patients survived for two years, compared to 81% of transplanted patients with active infections (28). In a study of 234 patients who underwent HSCT with a diagnosis of SCID in Türkiye between 1994 and 2014, the OS was 67.5%. Another study conducted in our clinic evaluated 72 patients with SCID who underwent HSCT between 1997 and 2017 (29). The study reported an overall survival (OS) rate of 80%. Both studies identified active infection during transplantation as the most significant negative factor affecting transplant outcomes (29, 30). Transplant success and OS rates are likely to be higher in patients who are diagnosed with SCID early with NBS and get isolation precautions and prophylaxis (28). BCG vaccination is routinely performed in countries with high Tuberculosis incidence. Vaccination time differs among countries. In Türkiye, a routine BCG vaccine is administered at two months. To prevent potential complications from BCG vaccination, it is crucial to diagnose SCID prior to vaccination. A recent study conducted by Bayram et al. in our clinic found that 38 out of 72 SCID patients were vaccinated with BCG before diagnosis, and BCG reactivation was observed in 26 out of 61 (42.6%) HSCT patients. The study found that BCG infection did not affect death in transplant patients, but disseminated BCG’itis was the most persistent complication in vaccinated patients during the post-transplant period. The inclusion of SCID in the NBSP will also prevent BCG vaccine-related complications in SCID patients (29). The mean age at the diagnosis of SCID was 5 months in Türkiye (30). In this study, both infants were diagnosed significantly earlier (at 4 weeks of age) because of NBS and were treated curatively without severe SCID-related infections, organ damages or routine BCG vaccination.

Various screening studies conducted in different countries and ethnic groups have reported different algorithms and TREC cutoff values. Additionally, these studies have found variable retest and new sample testing rates due to the use of different kits and methodologies. As a result of their outcomes over time, some countries have revised their TREC cutoff values. In Catalonia, the TREC cutoff values were reduced from 34 to 24 copies/µL for retests and 20 copies/µL for detection. Alarm cutoffs were established based on gestational age, with term newborns having 10 copies/µL or fewer and preterm newborns having ≤5 copies/µL. A second sample was requested if TREC values were below 20 copies/L. The retest rate decreased from 3.4% to 1.4%, and the rate of requiring a new sample decreased to 0.2%, while the rate of positive samples was 0.02% (25). A 5-year follow-up of NBS for SCID in Israel found that decreasing the TREC cutoff did not affect the identification of typical SCID cases, but it may reduce the number of different TCL instances detected. The study obtained different TREC cutoff values in the initial test and retest. The cutoff value was 17 copies/µL in the initial measurement, but it was accepted as 23 copies in the retest (in the sample taken from the 2nd card). The study reported retest and new sample test rates of 1.04% and 0.12%, respectively (31). In the Swedish study, all reanalyzed samples had a TREC result above 8.7 copies/well and remained above the mean reference TREC cutoff (TREC ≤6 copies/well) from the initial PCR analysis. The retest limit was reduced to 10 TREC copies per well, resulting in a decrease in the retest rate to 0.68% during the second period of the study (12). Based on our previous retrospective study with 5030 newborns, we established a TREC cutoff of 15 copies/µL. In this prospective study, the retest rate was 1.4%, the new sample rate was 0.58%, and the abnormal value rate was 0.049%. Although our TREC cutoff value and retest rates were comparable to the literature, our new sample test and abnormal test rates were higher. This highlights the need to establish a more precise cutoff point before requesting a new sample test and referring it for further evaluation due to an abnormal value. It is important to note that certain physiological factors, such as age and prematurity, can have an impact on TREC copy numbers (32, 33). Preterm infants require retests, new sample tests, and may receive false abnormal results. Some studies recommend determining separate cutoff values or monitoring those below (12, 19, 23, 34, 35). The research conducted in Wisconsin demonstrated that TREC values increase by 9.6% for every week of gestational age. Repeat DBS is required for preterm infants with low TREC results until discharge or until they reach 3 months of age (36). The study revealed that the median TREC values for term infants were significantly higher than those for premature infants. Very preterm infants (≥28-31 weeks) had lower TREC values compared to late preterm infants (≥32-37 weeks). Severely preterm babies had lower median TREC values. If SCID is included in Türkiye’s NBSP, a separate algorithm for new sample test cutoff values and preterm newborn assessment will be necessary.

The study found that newborns with low BW had lower TREC values compared to those with normal BW. Significant differences were observed in babies with BWs of ≤1500 g and 1501-2500 g. The difference was statistically significant for babies weighing ≥4001 g and ≤1500 g. TREC values were not affected by delivery mode or singleton or multiple births.

Additionally, the TRECs assay has the ability to detect other combined immunodeficiencies (CID) and syndromes that are associated with low levels of naive T cells. These include DiGeorge syndrome, trisomy 21, CHARGE syndrome, and ataxia-telangiectasia. The assay can also identify secondary causes of T-cell lymphopenia, such as cardiac defects, gastrointestinal abnormalities, multiple congenital defects, or prenatal steroid applications (32, 33). Kwan et al. conducted a study in the USA evaluating screening results of over 3 million babies. Of these, 52 babies were diagnosed with SCID, while 411 babies had conditions accompanied by T lymphopenia. Among the 411 non-SCID T-cell lymphopenia infants, 33% had a congenital syndrome associated with T-cell impairment. DiGeorge syndrome was the most common syndrome, accounting for 57%, followed by trisomy 21 at 15%. Other syndrome diagnoses included ataxia telangiectasia, trisomy 18, CHARGE syndrome, and other rare entities. Congenital heart disease was the most prevalent condition, followed by other medical conditions, vascular leakage, gastrointestinal anomalies, 4 cases of neonatal leukemia, and 12 cases of idiopathic T lymphopenia (6). In our study, we did not find any newborns with CID other than SCID, syndromic CID, or secondary or idiopathic lymphopenia. This could be due to the small sample size.

The literature reports that copy numbers of 0-3 TREC/µl are indicative of T cell lymphopenia (for SCID or other conditions) (37). In Massachusetts, 720038 newborns were screened over a period of ten years. All of the SCIDs detected, except for one with a TTC7A defect, had a TREC value of 0 (38). Two out of ten newborns in our study had 0 copies of TREC, while the remaining eight had TREC values of 12 and 14/µl. Two of remaining 8 infants were severely premature and had low BW. They died during the early newborn period, and one of them also had phenylketonuria. Therefore, further analysis could not be performed on them. For the remaining six infants, the TREC value changed from 12 to 14 copies/µl. Their detailed history, clinical and immunological examinations were normal. Immune work-up, including a CBC, analysis of immunoglobulin levels (IgG, IgA, IgM, total IgE) and PBLSs analysis, costs $30 per baby. Families are not required to pay any fee for these investigations, since the government covers all health costs of children under18 years by law in Türkiye. The families of the babies who were referred to our clinic for further immunological examinations were worried a little for a while. However, since our immunology laboratory is a reference laboratory all the tests were completed and resulted on the same day. So, all families of six suspected babies received normal immune work-up accordingly. The test results were shared immediately with the families that their concerns resolved completely.

After eliminating all possible causes of primary or secondary lymphopenia, these cases were considered false positives. The rate of abnormal values classified as ‘positive’ was 0.049% in our study, which required further immunological evaluation. This rate is comparable to the 0.04% reported in the French DEPISTREC study, but higher than the 0.02% rate reported in the Catalonia study (25, 39). In Türkiye, over 950000-1150000 babies are born every year. If SCID is included in NBSP screening, approximately 550 babies will require immunological evaluation each year. Cost calculations are reported as $4.25 in the USA and €4.21 in France. In general, worldwide costs are reported to be between $4-5 per test (3, 5, 39). In our study, the cost of TREC measurement was calculated as 5.1$ per test, which is approximately 150 TL (Turkish Lira). Delay in diagnosing SCID increases the frequency and severity of infections, leading to increased hospital admissions, intensive care unit admissions, and treatment costs (39). A cost analysis revealed that patients with delayed diagnosis incurred a health expenditure of at least 1.2 million TL (20). The NBSP for SCID is implemented as a cost-effective program that is both economically viable and meets a scientific need.

In conclusion, this study is the first in Türkiye to screen for SCID using TREC value in DBS. The study developed methods for SCID screening, including patient access plans, confirmation strategies, and treatments for positive patients. For early diagnosed SCID patients, genetic diagnosis and curative treatment were successfully performed. The incidence of SCID in Ankara and Konya provinces, identified as 1/10000 in this study, may be much higher across Turkey. Compared to many countries where SCID is included in the national NBSP, the incidence in Turkey is objectively higher. This study emphasizes the urgent need to include SCID into the national NBSP.

## Data Availability

The original contributions presented in the study are included in the article/**Supplementary Material**. Further inquiries can be directed to the corresponding author.
