# Supplementary material for: Newborn screening for SCID: the very first prospective pilot study from Türkiye
Source: Front Immunol. 2024 Oct 2;15:1384195. doi: 10.3389/fimmu.2024.1384195 (PMC11526446; doi:10.3389/fimmu.2024.1384195)
Supplement: Supplementary file 1 [file Table1.docx]

Supplementary Table 1. Sequences of the primers and probes

| **Name** | **Sequences** |
| --- | --- |
| TREC Forward primer | 5’ CACATCCCTTTCAACCATGCT 3’ |
| TREC Reverse primer | GCCAGCTGCAGGGTTTAGG |
| TREC_PROBE | 5’-FAM - ACACCTCTGGTTTTTGTAAAGGTGCCCACT - 3’TAMRA |
| B-Actin Forward primer | 5’ TCACCCACACTGTGCCCATCTACGAG 3’ |
| B-Actin Reverse primer | 5’ CAGCGAACCGCTCATTGCCATGG 3’ |
| B-ACTIN_PROBE | 5’-JOE - ATGCCCTCCCCCATGCCATCCTGCGT - 3’TAMRA |
