# Supplementary material for: Newborn screening for SCID: the very first prospective pilot study from Türkiye
Source: Front Immunol. 2024 Oct 2;15:1384195. doi: 10.3389/fimmu.2024.1384195 (PMC11526446; doi:10.3389/fimmu.2024.1384195)
Supplement: Supplementary file 2 [file Table2.doc]

| **Supplementary Table 2. Immune work-up of 6 infants with low Trec detected at initial NBS** | | | | | | | |
| --- | --- | --- | --- | --- | --- | --- | --- |
| **Infants (initials)** | **1**  **G.K.** | **2**  **E.B.** | **3**  **E.Y.** | **4**  **O.S. B** | **5**  **M.K.** | **6**  **Y.A.K.** | **Age references**  **mm^3^ (%)*** |
| **Province** | Ankara | Konya | Konya | Konya | Konya | Konya |  |
| **GW/BW** | 35 / 2800 | 35 / 2700 | 40 / 4700 | 40 / 3700 | 39 / 3350 | 41 / 3800 |  |
| **Age at blood tests (day)** | 29 | 38 | 60 | 40 | 40 | 28 |  |
| **ALC (mm^3^)** | 5100 | 3650 | 6120 | 3850 | 5410 | 9300 | 3000-10000 |
| **CD3+ T cells, mm^3^ (%)** | 3110 (61) | 2518 (69) | 4222 (69) | 2656 (69) | 3354 (62) | 6789 (73) | 2400-8100 (51-79) |
| **CD3+ CD4+ T cells, mm^3^ (%)** | 2142 (42) | 1606 (44) | 2940 (48) | 1617 (42) | 2488 (46) | 5115 (55) | 1400-5200 (31-54) |
| **CD3+CD8+ T cells, mm^3^ (%)** | 918 (18) | 912 (25) | 1285 (21) | 1001 (26) | 865 (16) | 1581 (17) | 600-3000 (11-33) |
| **CD19+ B cells, mm^3^ (%)** | 1326 (26) | 657 (18) | 1040 (17) | 655 (17) | 1677 (31) | 1209 (13) | 500-3600 (14-44) |
| **CD3-CD16+56+ cells, mm^3^ (%)** | 510 (10) | 182 (5) | 306 (5) | 500 (13) | 270 (5) | 837 (9) | 200-1800 (5-23) |
| **RTE, mm^3^ (%)** | 1456 (68) | 979 (61) | 1940 (66) | 950 (59) | 1368 (55) | 3887 (76) | 929-4981 (63-81) |
| **Immunoglobulin levels**  **(IgG, A, M)** | Normal | Normal | Normal | Normal | Normal | Normal | Normal |
| GW: Gestational week; BW: Birth weight (gr), ALC: Absolute Lymphocyte count, RTE: CD3+CD45RA+CD31+ cells  * Ikincioğullari A, Kendirli T, Doğu F, Eğin Y, Reisli I, Cin S, Babacan E. Peripheral blood lymphocyte subsets in healthy Turkish children. Turk J Pediatr. 2004 Apr-Jun;46(2):125-30. | | | | | | | |
